# Digging deeper into necrotizing enterocolitis: bridging clinical, microbial, and molecular perspectives

**DOI:** 10.1080/19490976.2025.2451071

**Published:** 2025-01-18

**Authors:** Deshuang Zhang, Dongke Xie, Yi Qu, Dezhi Mu, Shaopu Wang

**Affiliations:** a Department of Pediatrics, Key Laboratory of Birth Defects and Related Diseases of Women and Children (Ministry of Education), West China Second University Hospital, Sichuan University, Chengdu, China; b Division of Neonatology/Pediatric Surgery, Department of Pediatrics, The Affiliated Hospital of Southwest Medical University, Luzhou, China

**Keywords:** Necrotizing enterocolitis (NEC), preterm infants, microbes, dysbiosis, toll-like receptor 4 (TLR4), mechanism

## Abstract

Necrotizing Enterocolitis (NEC) is a severe, life-threatening inflammatory condition of the gastrointestinal tract, especially affecting preterm infants. This review consolidates evidence from various biomedical disciplines to elucidate the complex pathogenesis of NEC, integrating insights from clinical, microbial, and molecular perspectives. It emphasizes the modulation of NEC-associated inflammatory pathways by probiotics and novel biologics, highlighting their therapeutic potential. We further critically examine dysbiotic alterations within the gut microbiota, with a particular focus on imbalances in bacterial and viral communities, which may contribute to the onset of NEC. The intricate interactions among toll-like receptor 4 (TLR4), microvascular integrity, immune activation, and the inflammatory milieu are meticulously summarized, offering a sophisticated understanding of NEC pathophysiology. This academic review aims to enhance the etiological comprehension of NEC, promote the development of targeted therapeutic interventions, and impart the significant impact of perinatal factors on the formulation of preventive and curative strategies for the disease.

## Introduction

Necrotizing enterocolitis (NEC) is a well-documented and severe gastrointestinal disease that poses a significant threat to the lives of newborns. It is particularly prevalent among preterm infants, with an incidence rate of approximately 10% in those weighing less than 1500 g at birth.^1,2^ NEC is characterized by intestinal inflammation and necrosis, predominantly affecting the terminal ileum and proximal colon. In infants with NEC, the compromised intestinal barrier function facilitates the translocation of enteric organisms into the bloodstream, which often leads to severe sepsis and, in many cases, results in mortality.^3,4^ Notably, a substantial proportion of infants with NEC (ranging from 24% to 39%) are accompanied by sepsis concurrently.^5,6^ The mortality rate of preterm infants with NEC varies from 20% to 30%^1,7,8^ and can reach up to 50% in extremely low body weight (ELBW, birth weight <1000 g) infants.^9^ Moreover, surviving infants from NEC often experience enduring complications, such as short bowel syndrome, intestinal strictures, growth delay, and neurodevelopmental impairments.^10–12^

The initial medical interventions for NEC involve halting all oral feedings, nasogastric decompression of the gut, administering broad-spectrum antibiotics, and providing additional fluids intravenously. Surgical intervention is typically recommended for infants with a deteriorating condition, particularly in instances of bowel perforation.^13^ Despite several decades of clinical experience in managing NEC infants, the overall mortality rates and treatment strategies have remained largely unaltered since the initial characterization of the disease many years ago.^13,14^ However, significant progress has been achieved in the last decade regarding the comprehension of microbial and molecular mechanisms underlying the pathogenesis of NEC. These advancements offer the potential for enhancing the development of preventive and therapeutic approaches to mitigate the severe repercussions associated with this disease. Therefore, this review aims to examine NEC from a clinical perspective and, more significantly, to synthesize the most recent discoveries from the microbial and molecular insights.

## Clinical perspective

**Figure 1.**
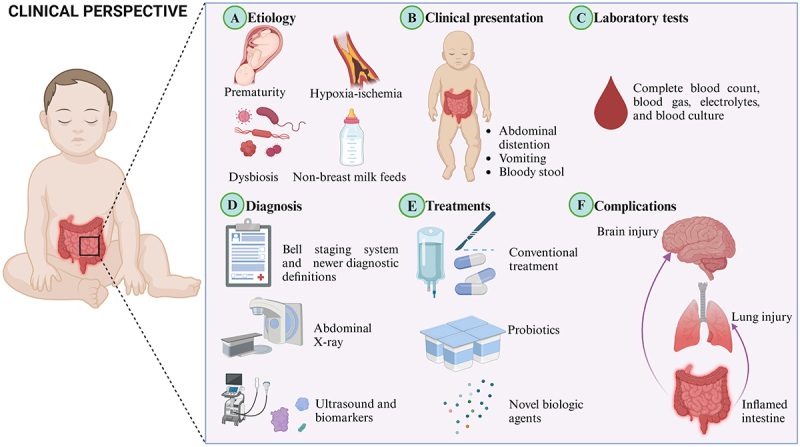
Clinical perspective of NEC. (a) Etiology. NEC is a multifactorial disease. Prematurity, dysbiosis, hypoxia-ischemia, and non-breast milk feeds are common factors contributing to the development of NEC. (b) Clinical presentation. Abdominal distention, vomiting, and bloody stool are typical signs of NEC. (c) Laboratory tests. Complete blood count, blood gas, electrolytes, and blood culture are routine tests of NEC. (d) Diagnosis. Bell staging system, together with clinical manifestations and abdominal X-ray findings are usually used to diagnose and assess the severity of NEC. In response to the limitations of bell criteria, multiple newer diagnostic definitions of NEC have been proposed. Ultrasound and biomarkers remain an ongoing pursuit. (e) Treatments. Conventional treatment includes halting all oral feedings, performing nasogastric decompression, administering broad-spectrum antibiotics, providing supplemental fluids intravenously, and surgical intervention if necessary. Probiotics in protecting against NEC is an enthralling and burgeoning area, and novel biologic agents continue to be a compelling frontier in medical research. (f) Complications. Secondary organ involvement, particularly lung and brain injuries, is commonly observed in addition to the primary intestinal effects.

NEC carries substantial morbidity and mortality. Here, we delve into the multifaceted clinical perspective of NEC, encompassing its etiology, clinical presentation, diagnosis, laboratory tests, treatments, and potential complications (Figure 1). The following subsections will dissect the complex clinical landscape of NEC, offering insights into the current understanding and future directions in the care of infants suffering from this disease.

### Etiology

The underlying pathogenesis of NEC is still not completely understood due to the complex array of causative elements involved. Although it is generally hypothesized that the interruption of the blood supply to the intestinal mucosa causes the local hypoxia and ischemia and impairs intestinal peristalsis,^15^ the precise factor that initiates NEC is not clarified. The inflammatory damages culminating in hemorrhage, erosion, and necrosis of the mucosa then begin to occur, which may be associated with multiple factors, including prematurity, dysbiosis, hypoxia-ischemia, and non-breast milk feeds.^16^ Moreover, a multicenter study comparing concordance rates in monozygotic and dizygotic twins suggests that 51.9% of the variance in susceptibility to NEC can be explained by genetic and shared environmental factors, indicating the potential role of genetics in predisposing the infant to NEC.^17^ Other important clinical factors implicated in NEC include the transfusion of red blood cells (RBC), the administration of H_2_ blockers, and the long-term use of antibiotics.^18^ Interestingly, a human study observed that severe anemia, as opposed to RBC transfusion, was associated with a higher risk of NEC in very low birth weight (VLBW, birth weight <1500 g) infants.^19^ Subsequently, with the mouse model, researchers observed severe anemia induced a low-grade inflammatory state within the intestinal mucosa, evidenced by macrophage infiltration.^20^ Subsequent RBC transfusions activated these macrophages through a toll-like receptor 4 (TLR4)-mediated mechanism, resulting in bowel injury analogous to NEC. This study further revealed that RBC transfusions may initiate NEC-like bowel injury in mouse pups that experienced anemia, but this effect was not observed in non-anemic mice.^20^ Thus, it may suggest that the anemia, rather than the transfusion itself, is the critical factor for the intestinal injury. Certainly, ongoing research is still actively seeking to identify additional factors and underlying processes that contribute to this devastating disease.

### Clinical presentation

NEC mainly affects preterm infants and often manifests after the onset of feeding, with most cases occurring between the second and third week of life. Notably, there is an inverse relationship between gestational age or birth weight and the occurrence of NEC. In more mature preterm infants, NEC appears to occur as early as the first week of postnatal; whereas in ELBW preterm infants, it can be delayed up to an average of 32 days after birth.^21^ In general, the progression of NEC appears to reach its peak at the 31st week of gestational age.^22^ NEC presents with diverse and often nonspecific clinical spectrums, which include subtle signs, including feeding intolerance, inadequate response to stimuli, lethargy, fluctuating body temperature, and apnea.^23^ This variability complicates early differentiation from other conditions, notably sepsis.^24,25^ Common signs of NEC include feeding intolerance, inadequate response to stimuli, lethargy, fluctuating body temperature, and apnea. Prompt recognition and treatment are crucial, as failure to intervene in time may result in rapid progression to typical signs such as abdominal distention, vomiting, and bloody stool. A recent retrospective cohort study found that a significant proportion (32%) of cases of medical NEC progressed to surgical NEC within three days, although in some it presented as surgical NEC from the outset.^26^ This surgical stage condition is often accompanied by subsequent multiple organ systems dysfunction.

### Diagnosis

To date, the Bell staging system remains the most commonly used clinical definition for NEC. It is specifically designed for evaluating the severity of NEC and should not be misconstrued as a diagnostic tool in clinical practice. It categorizes the stages of NEC and is usually used together with the clinical manifestations and abdominal X-ray findings to complement the diagnosis of the disease.^27^ Its clear descriptive nature has made it a widely accepted tool in the medical community, which classified NEC into three stages according to this system, namely stage I (suspected NEC), stage II (confirmed NEC), and stage III (advanced NEC). Specific radiographic signs, such as pneumatosis intestinalis or portal venous gas, confirm the diagnosis of NEC at least until stage II. In stage I, the clinical manifestations are characterized by nonspecific signs, such as gastric retention, feeding intolerance, or mild abdominal distension.^27^ Meanwhile, plain abdominal radiographs may show normal findings, intestinal dilation, or subtle signs of intestinal obstruction.^27^ However, the improper use of this system has, unfortunately, led to instances of overdiagnosis of NEC. Given that the observations in stage I of NEC are not unique, they can be challenging to differentiate from signs in very preterm infants and other medical conditions, including cow’s milk sensitivity, food protein intolerance, polycythemia, and early-stage sepsis.^3^ Moreover, the clinical presentation of spontaneous intestinal perforation (SIP) closely resembles that of NEC. Based on the Bell criteria, many infants with SIP could be classified as Bell stage III, but in fact, SIP differs from NEC.^28^ In response to these limitations, multiple newer diagnostic definitions of NEC have been proposed,^29^ including the original modification of Bell criteria (ModBell), Vermont Oxford Network (VON), Centers for Disease Control (CDC), United Kingdom (UK), Two out of 3 (2 of 3), Stanford (ST), and International Neonatal Consortium (INC). Lueschow et al. conducted a comprehensive comparative analysis of current NEC definitions, employing both standard statistics and six supervised machine learning classifiers.^30^ Results suggested that current definitions of NEC showed superior performance in terms of sensitivity (0.826 for INC) and specificity (0.969 for ST). In contrast, the ModBell (IIA+) staging revealed a moderate sensitivity (0.783) but a low specificity (0.531). However, this is just a preliminary study from a retrospective analysis at a single center and should be explored in more depth with a larger and more diverse cohort of cases. Concurrently, an ongoing pursuit is developing with more precise diagnostic criteria, advances in abdominal ultrasound, and specific gut-associated biomarkers.^31,32^

### Laboratory tests

Laboratory tests, while often nonspecific, have certain limitations in their diagnostic utility. Despite these constraints, they can reveal abnormalities such as leukocytosis or leukopenia, neutropenia, anemia, thrombocytopenia, elevated C-reactive protein (CRP), metabolic acidosis, electrolyte disturbance, etc. Importantly, among these abnormalities, some are particularly noteworthy for their substantial diagnostic and prognostic implications. Leukocytosis greater than 30 × 10^9^/L, a pH level below 7.25, and an increase in blood glucose of 1.5 mmol/L or more within 24 hours may indicate the development of NEC with intestinal perforation.^33^ A minority of infants may have positive blood cultures, which are dominantly attributed to gram-negative bacteria.^34^ Thrombocytopenia (platelet count < 150 × 10^9^/L) is very common in the context of NEC. Almost 50–95% of infants with NEC develop thrombocytopenia within the initial 24 to 72 hours after diagnosis.^35^ In infants with a confirmed diagnosis of NEC, the severity and extent of bowel injury appear to be more closely correlated with thrombocytopenia. A rapid drop in platelet count to less than 50 × 10^9^/L – a sign of severe thrombocytopenia – within 24 hours of NEC diagnosis typically indicates intestinal necrosis and the need for surgical intervention.^36^ Meanwhile, severe thrombocytopenia is a predictive indicator of mortality in preterm infants with NEC.^37^ Neutropenia, defined as an absolute neutrophil count (ANC) less than 1500/μL, is commonly observed in the severe stage of NEC and is associated with poor outcomes. A trend indicates that neutropenia is more frequently observed among non-survivors of NEC compared to survivors.^38^ Even in a newer retrospective cohort study involving 157 infants with NEC, a reduction in ANC upon NEC onset correlated with a higher likelihood of developing severe surgical NEC (odds ratio [OR] 1.25; 95% CI 1.11–1.41).^39^ Intriguingly, in preterm infants following NEC, gestational age-specific hematological profiles are observed. In lower preterm infants (born below 28 gestational weeks), thrombocytopenia independently predicts the need for surgery. In infants aged 28 to 32 gestational weeks, persistent thrombocytopenia, lymphopenia at 72 hours, and elevated CRP levels 48 hours after the onset of NEC are indicative of the need for surgical intervention. Conversely, in more mature preterm infants (born at 32 gestational weeks or more), severe thrombocytopenia does not serve as a predictor of surgical intervention. Yet, elevated CRP levels 72 hours after the onset of NEC predict surgery in this population.^40^ Even more, some researchers use a combination of multiple laboratory indicators to differentiate surgical or death NEC from medical NEC.^41,42^ However, current studies are mainly retrospective and limited to single-center studies. Future studies should be well-designed, prospective, and multicenter to determine the role of hematologic biomarkers in the early diagnosis and prognosis of NEC.

In recent years, numerous studies have investigated blood, fecal, and urine biomarkers for NEC, intending to enable timely identification and diagnosis through their inflammatory profiles.^43^ Common nonspecific markers include serum amyloid A (SAA), serum levels of C5a, transforming growth factor (TGF)-β, and levels of IL-1β, IL-6, IL-8, and IL-10,^44^ all of which play important roles in the pathogenesis of NEC. Higher levels of SSA, C5a, IL-1β, IL-6, IL-8, and IL-10, and lower levels of TGF-β are usually found in preterm infants with NEC.^45,46^ In parallel, combined serum IL-6 and IL-8 levels have been indicated as more reliable indicators for NEC stages II/III; IL-8 could be used to predict surgical NEC; and serum C5a levels were more accurate than other markers in predicting mortality and the need for surgery upon NEC diagnosis.^46–48^ However, these markers lack specificity and can only partially distinguish NEC from other inflammatory diseases.

Frequent specific markers contain serum cytosolic β-glucosidase (CBG), intestinal fatty acid-binding proteins (I-FABP), and fecal calprotectin, which may serve as early diagnostic indicators for the development of NEC and the evaluation of its severity. A study enrolling 192 preterm infants without NEC and 13 preterm infants with NEC revealed that the cutoff value of 15.6 mU/mg of serum CBG could discriminate infants having NEC with 84.6% sensitivity and 85.9% specificity.^49^ Two meta-analyses revealed that I-FABP levels had high specificity in plasma (91%) and urine (73%), yet the moderate sensitivity of 64% in both media constrains its reliability as a NEC biomarker.^50,51^ In a meta-analysis including 10 studies of 568 infants with NEC, the sensitivity and specificity of fecal calprotectin were 0.91 (95% CI 0.82–0.97) and 0.93 (95% CI 0.88–0.96) for four studies focusing on preterm infants, and 0.86 (95% CI 0.77–0.92) and 0.94 (95% CI 0.90–0.97) for five studies defined stage II or above of NEC.^52^ However, a large inter- and intra-individual variability was observed in preterm infants, which may limit the value of fecal calprotectin for NEC prediction. In addition, emerging biological techniques, including proteomics for large-scale protein analysis and metabolomics for comprehensive metabolic profiling,^32^ offer the potential to enhance the accuracy of NEC screening. Despite these advancements, further clinical and basic research is essential to validate the efficacy of specific biological markers or combinations of diagnostic indicators.

### Treatments

The routine medical interventions for NEC include halting all oral feedings, performing nasogastric decompression, administering broad-spectrum antibiotics, and providing supplemental fluids intravenously.^24,53^ Surgical intervention is indicated in some conditions, particularly those that have progressed with bowel perforation.^13^ Despite extensive research and decades of dedicated efforts, a specific and universally effective treatment for NEC remains elusive. Nevertheless, with an advanced understanding of the mechanisms contributing to NEC, a variety of innovative potential therapeutic strategies for NEC have been discovered in recent years, for example, probiotics and a series of novel biologic agents.

#### Probiotics

The role of probiotics in protecting against NEC is an enthralling and burgeoning area in medical research. Numerous trials have illuminated their potentially preventative benefits for NEC.^54^ A meta-analysis, involving 45 trials and 12,320 participants, indicated that the concurrent administration of *Bifidobacterium* and *Lactobacillus* is linked to a significant reduction in both mortality rates (risk ratio [RR] 0.56; 95% CI 0.34–0.84) and NEC morbidity (RR 0.47; 95% CI 0.27–0.79), as opposed to the use of placebo supplements.^55^ Also, an interesting finding from this study revealed that the *Bifidobacterium*-prebiotic combination had the highest likelihood of achieving the lowest mortality rate, whereas the *Lactobacillus*-prebiotic combination was most likely to achieve the lowest NEC rate. Similar results were reported in another systematic review and network meta-analysis of 63 trials involving 15,712 preterm infants.^56^ Interventions with moderate- or high-quality evidence significantly reduced the incidence of severe NEC (stage ≥ II), including the combination of one or more species of *Lactobacillus* and *Bifidobacterium* (OR 0.35; 95% CI 0.20–0.59), with efficacy observed for *Bifidobacterium animalis species lactis* (OR 0.31; 95% CI 0.13–0.74), *Lactobacillus reuteri* (OR 0.55; 95% CI 0.34–0.91), and *Lactobacillus rhamnosus* (OR 0.44; 95% CI 0.21–0.90). However, a more recent Cochrane Systematic Review indicates that the effects of probiotic supplements on NEC risk, as well as morbidity and mortality among very preterm or VLBW infants, range from low to moderate certainty. In extremely preterm or ELBW infants – the highest risk population for NEC, probiotic supplements even had little or no effect on preventing NEC (RR 0.92; 95% CI 0.69–1.22).^57^ Taken together, some meta-analyses of probiotics studies have shown benefits, yet they are not considered to be strong studies based on the massive heterogenicity of study protocols and study bacteria used. Conflicting data presents its efficacy in VLBW infants, especially ELBW infants, who are at highest risk for NEC. Another important concern against probiotic supplements is the risk of causing sepsis in preterm infants, also named probiotic sepsis. Probiotic sepsis, a severe sometimes life-threatening complication in preterm infants, is defined as positive blood or cerebrospinal fluid (CSF) cultures that isolate the strain of the administered probiotic, accompanied by clinical signs of infection.^58^ While uncommon, reports from various investigators detailed individual cases or case series of sepsis attributed to probiotic supplementation in preterm infants.^59–61^ In a recent systematic review, 16 reports of 32 preterm infants diagnosed with probiotic sepsis were evaluated, with most cases born under 32 gestational weeks. The predominant microorganisms were *Bifidobacterium* (*n* = 19), followed by *Lactobacillus* (*n* = 10), and *Saccharomyces* (*n* = 3). Two studies each reported one neonatal death and 12 neonates had comorbidities.^58^ Therefore, the efficiency and safety due to the administered probiotic strains are all potential barriers against the prophylactic use of probiotics in preterm infants. Of particular note is the American Academy of Pediatrics (AAP) statement against probiotic use and the regulatory ban by the US Food and Drug Administration (FDA). These authoritative positions emphasize the need for heightened vigilance and further research on the use of probiotics for this vulnerable population.

The prophylactic mechanisms acting by probiotics against NEC encompass a complex interplay of biological processes, which are mainly validated through *in vitro* studies and animal models. In animal models, administration of probiotics enriched with bacterial DNA, particularly CpG DNA motifs, leads to the activation of Toll-like receptor 9 (TLR9), which serves as a negative regulator of the TLR4 signaling cascade in enterocytes, thus reducing the lipopolysaccharide (LPS)-mediated TLR4 signaling.^62,63^ This modulation of the immune response is thought to attenuate the severity of NEC. Meanwhile, the bacterial DNA was observed to significantly diminish the levels of proinflammatory signaling in cultured enterocytes as well as *in vitro* samples of resected human ileum, suggesting the potential therapeutic efficacy of the probiotic in the clinical management of NEC.^63^ Liu et al. have shown that the early introduction of *Lactobacillus reuteri* to newborn mice effectively prevented NEC, with the preventative effect mediated by an increase in the intestinal Foxp3+ regulatory T cell (Treg) population and the concurrent stimulation of beneficial gut microbiota proliferation.^64^ Intriguingly, *in vitro* studies, probiotic culture media (PCM) serve multiple functions in the context of NEC. PCM mitigates the risk of NEC by modulating immune response genes, particularly within the NF-κB signaling pathway, which is crucial for regulating inflammation.^65^ Furthermore, PCM helps to maintain the intestinal barrier’s integrity by normalizing the tight junction protein expression and reducing the expression of extracellular matrix (ECM) remodeling genes, providing important protection against NEC.^65,66^ Also, PCM’s capacity to decrease the production of inflammatory cytokines IL-6 and IL-8 contributes to its anti-inflammatory effects.^65,67^

#### Novel biologic agents

Novel biologic agents for NEC continue to be a compelling frontier in medical research, driven by the potential to transform treatment strategies for this devastating disease (Table 1).

**Table 1.** Novel biologic agents with therapeutic potential for NEC.

Novel biologic agents	Mechanism	Consequence	Experimental approach (Reference)
C34	Inhibit TLR4; suppress LPS signaling	Reduce systemic inflammation	*In vitro* study (human sample, cell culture) and animal model (mice)^68,69^
MDP	Activate NOD2 downregulate apoptosis regulatory protein SMAC-diablo reduce enterocyte apoptosis inhibit TLR4 signaling	Prevent NEC	*In vitro* study (cell culture) and animal model (mice)^70^
I3C or “A18”	Activate AHR reduce TLR4 signaling	Prevent NEC	*In vitro* study (human sample, and cell culture) and animal model (mice)^72,73^
J11	Modulate BDNF secrete suppress TLR4-mediated pro-inflammatory signaling	Attenuate the severity of NEC	Animal model (mice)^74^
Arginine; HB-EGF, sodium nitrate, sildenafil, and nicotinamide riboside	Increase eNOS improve intestinal microcirculation	Reduce NEC severity	Human study^75^ and animal models (rats, mice)^76–78^
Stem cells, specifically ISCs, MSCs, and NSCs	Anti-inflammatory, anti-apoptotic, and gut barrier-enhancing effects	Improve blood supply and protect against NEC	Human study^80^; in *vitro* study (human sample, organoid, and cell culture) and animal model (rats, mice)^79^
Exosomes, from stem cells and breastmilk	Delivery anti-inflammatory cytokines and miRNAs modulate immune response; Promote epithelial cell proliferation and differentiation enhance intestinal barrier function; Activate Wnt/β-catenin signaling and influence TLRs expression attenuate the inflammatory cascade	Tissue repair and ameliorate NEC	*In vitro* study (human sample, cell culture) and animal model (rats, mice)^87–91^
ATRA	Restore lymphocyte homeostasis toward Tregs	Reduce NEC severity	Animal model (rats)^92^
Anti-TNF-α antibodies	Lower levels of TNF-α and IL-18; Reduce intestinal wall permeability; Express pro-apoptotic markers such as Bax and cleaved caspase-3	Alleviate intestinal damage and a lower incidence of NEC	Animal model (rats)^93^
Tocilizumab	Inhibiting IL-6-mediated signal transduction	Reduce the severity of NEC	Animal model (rats)^94^
Celecoxib	Decrease pro-inflammatory cytokines TNF-α and IFN-γ, elevate anti-inflammatory cytokine IL-10 mitigate inflammation; Reduce TOS and MDA, enhance TAS, SOD, and GPx activity ameliorate oxidative stress; Modulate caspase-3 activity diminish epithelial cell apoptosis	Protect against NEC	Animal model (rats)^95^
ANGPT2	Knockdown ANGPT2 block Notch signaling mitigate LPS-induced inflammation, barrier dysfunction and ER stress	A potential therapeutic target for NEC	*In vitro* study (cell culture)^96^
FMT or FFT	Transfer microbial community or microbial filtrate into recipients	Prophylaxis and therapeutic intervention for NEC	Animal models (mice, pigs)^97,98^

C34, a novel inhibitor of TLR4 that is active both *in vitro* and *in vivo* models. *In vitro* studies, C34 has been suggested to inhibit TLR4 in enterocytes and macrophages. In mouse model of NEC, C34 effectively reduced systemic inflammation.^68^ Another *in vitro* study showed that C34 suppresses LPS signaling in human ileum tissue resected from NEC infants.^69^ Therefore, C34 possesses significant potential as a therapeutic agent for treating TLR4-mediated inflammatory conditions such as NEC. Similarly, *in vitro* and animal model studies, the use of muramyl-di-peptide (MDP), an agonist of nucleotide-binding-oligomerization domain-2 (NOD2), activates NOD2 and results in the downregulation of the apoptosis regulatory protein SMAC-diablo. This in turn indirectly inhibits the TLR4 signaling pathway by reducing enterocyte apoptosis, contributing to the prevention of NEC.^70^ In a large human study enrolling 9,082 VLBW infants, carriers of two or more NOD2 loss-of-function mutations had a significantly increased risk for NEC requiring surgery, highlighting a potential protective role of NOD2 in the development of NEC.^71^

Some investigators found the expression of AHR is significantly reduced in both *in vitro* studies and animal models.^72,73^ In particular, the use of AHR ligands indole-3-carbinol (I3C) or the clinical compound “A18” during pregnancy or of breast milk, effectively activates AHR of the intestine and prevents NEC in the offspring. This activation is associated with a reduction in TLR4 signaling. Meanwhile, the study suggests that breast milk may contribute to the prevention of NEC by activating AHR, although further research is needed to clarify the specific contribution of AHR ligands in breast milk. In animal model, Kovler et al. identified a small molecule, J11, which modulates enteric glia to secrete brain-derived neurotrophic factor (BDNF). Through augmenting BDNF release, J11 effectively suppresses TLR4-mediated pro-inflammatory signaling, thereby attenuating the severity of NEC.^74^ Also *in vitro* study application of J11 to human intestinal resection specimens of NEC demonstrated a significant reduction in inflammation.

Intestinal ischemia, resulting from reduced endothelial nitric oxide synthase (eNOS) activity in the mesenteric endothelium, is a central pathogenic factor in the development of NEC. Based on this finding, an array of agents has emerged as promising candidates for NEC treatment. In a systematic review enrolling 285 preterm infants, it was found that arginine supplementation significantly reduced the risk of developing NEC compared to the placebo group (RR 0.38; 95% CI 0.23–0.64).^75^ From this insight, a beneficial effect on intestinal microcirculation and a reduction in NEC severity have been observed in animal models with the use of other agents such as heparin-binding EGF-like growth factor (HB-EGF), sodium nitrate, sildenafil, and nicotinamide riboside.^76–78^

Given the essential role of stem cells in protecting the gut, stem cell therapy has become a focus of NEC research. Significant progress has been made in animal models, demonstrating the protective potential of stem cells through their anti-inflammatory, anti-apoptotic, and gut barrier-enhancing effects against NEC.^79^ Specifically, intestinal stem cells (ISCs), mesenchymal stem cells (MSCs), and neural stem cells (NSCs) are most commonly used for the treatment of NEC. Building on these findings, a clinical application of stem cell therapy in 2019 marked a significant milestone.^80^ In this study, a full-term baby aged 22 days developed NEC 14 hours after cardioversion for supraventricular tachycardia (SVT). This infant underwent laparotomy, confirming the diagnosis of pan-NEC, and 60 cm of the necrotic and perforated bowel was resected. On the fourth day of postoperative, this baby received an intravenous infusion of MSCs to restore and rescue the remaining intestine. After the application of MSCs, there was a significant improvement in blood supply to the mesentery, and the infant did not develop short bowel syndrome in the subsequent nearly one-year follow-up period. Thus, MSCs may be a promising therapy for ischemic bowel segments in NEC, potentially preventing short bowel syndrome. However, this is just a case report and the infant also had a resection of the necrotic bowel, which may have contributed to the observed improvement independently of the stem cell therapy.

It is well-recognized that breast milk serves as a potent prophylactic agent against NEC among preterm infants. This is attributed to the presence of numerous bioactive components, like immunoglobulin A (IgA),^81^ human milk oligosaccharides (HMOs),^82^ exosomes,^83^ epidermal growth factor (EGF),^84^ arginine,^75^ sodium nitrate,^77^ lactoferrin^85^ and glycomacropeptide,^86^ which play a crucial role in neonatal health and disease prevention. The mechanisms of action of most of these bioactive components are explained in other sections of the article, and some have been validated in human studies, such as IgA, HMOs, arginine, and lactoferrin.

Exosomes, particularly those derived from stem cells and breast milk, are emerging as potent alternatives to stem cell transplantation due to their minimal immunogenicity, lower ethical concerns compared to the use of stem cells, and their potential for targeted delivery, offering a safe and effective means to modulate tissue repair and regeneration in NEC treatment.^87^ *In vitro* studies and animal models have demonstrated their protective effects against NEC through multiple mechanisms, including modulating immune responses, enhancing intestinal barrier function, activating the Wnt/β-catenin signaling pathway, and influencing the expression of TLRs.^87–91^

Additionally, several therapeutic options have shown promise predominantly in preclinical animal models. All trans-retinoic acid (ATRA), a synthetic form of retinoic acid, is the active metabolite of vitamin A. Dietary supplementation with ATRA could help balance immune cells, specifically by increasing the number of regulatory T cells (Tregs) relative to inflammatory T helper 17 cells (Th17). This balance was also shown to significantly reduce the severity of NEC in a rat pups model.^92^ Tumor necrosis factor-alpha (TNF-α), a significant mediator in NEC development, has been targeted in therapeutic approaches. A previous study observed that the administration of anti-TNF-α antibodies to neonatal rats model with NEC significantly lowered hepatic and intestinal levels of TNF-α and IL-18.^93^ This intervention also reduced intestinal wall permeability and the expression of pro-apoptotic markers such as Bax and cleaved caspase-3. Tocilizumab, a humanized anti-IL-6 receptor monoclonal antibody, demonstrated therapeutic efficacy in reducing the severity of NEC in newborn rats model by specifically inhibiting the IL-6-mediated signal transduction pathway, suggesting that tocilizumab may offer a potential therapeutic approach for NEC management.^94^ Celecoxib, a selective COX-2 inhibitor, has demonstrated protective effects against NEC in neonatal rats model.^95^ The compound mitigates inflammation by decreasing levels of pro-inflammatory cytokines TNF-α and IFN-γ, while concurrently elevating the anti-inflammatory cytokine IL-10. Additionally, celecoxib ameliorates oxidative stress by reducing total oxidant status (TOS) and malondialdehyde (MDA) levels, and enhancing total antioxidant status (TAS), superoxide dismutase (SOD), and glutathione peroxidase (GPx) activity. Furthermore, celecoxib diminishes ileal epithelial cell apoptosis, potentially through modulation of caspase-3 activity, thereby improving pathological outcomes. Angiopoietin-2 (ANGPT2), a member of the major angiogenic growth factors, is implicated in vascular remodeling. In an *in vitro* study,^96^ researchers stimulated intestinal epithelial IEC-6 cells with lipopolysaccharide (LPS) to induce a NEC model. They found that the knockdown of ANGPT2 significantly mitigated LPS-induced inflammation, barrier dysfunction, and endoplasmic reticulum (ER) stress in these cells by blocking the Notch signaling pathway. This finding suggests that ANGPT2 represents a potential therapeutic target for the treatment of NEC.

In addition, both fecal microbiota transplantation (FMT) and fecal filtrate transplantation (FFT) exhibit potential in the prophylaxis and therapeutic intervention for NEC.^97,98^ FMT facilitates the transfer of a complete microbial community from a healthy donor directly into the recipient’s intestinal milieu.^99^ Conversely, FFT involves the transference of a microbial filtrate that has undergone micropore filtration to eliminate bacteria, potentially retaining bacteriophages and other soluble factors. Unlike FMT, FFT presents a potentially safer alternative due to the mitigated risk of transferring pathogenic microbes, an inherent concern with FMT, while preserving the beneficial modulation of the gut microbiota. Hence, FFT is posited as a promising treatment modality for the vulnerable preterm infant population. However, these two potential treatments for NEC are currently focused on experimental animal models.

### Complications

While NEC primarily affects the gastrointestinal tract, emerging evidence indicates its systemic effects on secondary organs like the lungs, brain, liver, and kidneys.^100,101^ Pulmonary and neurological complications, also termed NEC-associated lung injury and NEC-associated brain injury, are especially notable, impacting up to 70% of the infants who survive the condition.^102^

Lung disease usually develops more severely in preterm infants with NEC than those without NEC.^103^ In infants with severe NEC needing surgery, about 25% also suffer from severe lung disease.^104^ The lungs are particularly vulnerable to injury in the context of NEC due to the complex interactions between the gastrointestinal and respiratory tracts. These interactions occur across multiple factors, including the microbiota, immune responses, and metabolites,^105^ but the specific mechanism focuses on *in vitro* studies and animal models. A significant consequence of NEC on the pulmonary system is the development of pulmonary inflammation. In a neonatal rat model conducted one day after the onset of NEC, it was observed that the affected group showed inflammatory cell infiltration in the lung epithelium and interstitium, accompanied by the presence of inflammatory exudates in the alveolar spaces and bronchi.^100^ On day three, persistent thickening of the alveolar walls and marked interstitial edema were observed. These conditions have been shown to gradually decrease over time. In a newborn mice NEC model, Jia et al. found that NEC-associated lung injury was partly due to the activation of TLR4 on the lung epithelium because the deletion of TLR4 from the pulmonary epithelium confers protection to the lungs. Mechanistically, activation of TLR4 on intestinal epithelium triggers the release of high-mobility group box 1 (HMGB1) from the intestine. This, in turn, activates pulmonary epithelial TLR4, resulting in the upregulation of the neutrophil-recruiting chemokine C-X-C motif ligand 5 (CXCL5) and subsequent infiltration of pro-inflammatory neutrophils into the lung tissue.^106^ Meanwhile, the authors observed this mechanism *in vitro* using human bronchiole epithelial cell lines. Activation of TLR4 also induces expression of C-C motif chemokine ligand 25 (CCL25) on pulmonary epithelial, resulting in the recruitment of pro-inflammatory Th17 cells to the lungs, leading to lung injury after NEC. A similar result was found *in vitro* study from the human lung sample of infants with NEC.^107^ Moreover, the inflammatory reaction in the lungs leads to a reduction in eNOS, which would reduce blood flow and aggravate the lung injury.^108^ Importantly, lung injury resulting from NEC often leads to the development of bronchopulmonary dysplasia (BPD),^109^ a chronic lung disease frequently encountered in preterm infants. In a retrospective longitudinal study involving 250 NEC survivors and 2,909 matched control subjects, the risk of developing BPD was significantly higher in the NEC group over a long period. Specifically, the adjusted OR compared to matched controls was 3.0 (95% CI 1.8–5.6) in the medical NEC group and 4.0 (95% CI 2.0–7.0) in the surgical NEC group at 6–12 months of follow-up and 4.6 (95% CI 1.4–15.0) and 5.5 (95% CI 2.0–16.0) at 24–36 months of follow-up, respectively.^103^ The inflammatory response triggered by NEC contributes to the pathogenesis of BPD, leading to abnormal lung growth and impaired pulmonary function. Furthermore, mechanical ventilation and oxygen therapy, often essential for NEC management, can potentially lead to an aggravation of lung injury, which is thought to be a significant factor in the development of BPD.^103, 110^

The brain, a complex and vital organ, is central to a range of critical functions, including cognition, sensation, and motor control. Although all preterm infants are at risk of neurodevelopmental impairment, those with NEC face a particularly high risk. Neurodevelopmental outcome data from 18 to 22 months of age in the ELBW population indicates that infants with NEC are more likely to have a Psychomotor Development Index (PDI) <70 compared to infants without NEC (OR 2.64; 95% CI 1.18–5.91).^111^ In a systematic review of 7,843 VLBW children, the median follow-up period was 20 months (range 12 to 156). Neurodevelopmental disorders occur in 45% of children with NEC compared to 35% of children without NEC (OR 1.58; 95% CI 1.25–1.99).^112^ NEC can cause marked alterations in brain structure and function. A common observation is white matter injury,^113,114^ which presents as disruptions in myelination, axonal damage, and gliosis. The severe and enduring alterations in brain structure and function can lead to substantial neurological consequences, such as cognitive and motor impairments, learning disabilities, and behavioral disorders. A systematic review observed a significant association between NEC and markedly poorer neurodevelopmental outcomes.^112^ Moreover, the progression to advanced NEC and the requirement for surgical intervention significantly increase the risk of neurological impairment. The mechanisms by which NEC affects the brain are complex and not fully understood. It is proposed that the systemic inflammation triggered by NEC initiates a cascade of events that can ultimately lead to brain injury. Inflammatory mediators can activate TLR4 signaling and establish direct neural communication pathways, including those within the enteric nervous system (ENS) and via the vagus nerve.^115^ This activation may lead to white matter injury and compromised myelination. In NEC, damage to the intestinal barrier allows bacteria and their metabolites to leak into the bloodstream. This can lead to a widespread infection throughout the body and may trigger a series of inflammatory responses in the nervous system.^116^ Current studies suggest several potential mechanisms linking NEC to brain injury. It highlights systemic hypoperfusion,^117^ which can lead to reduced blood flow in the brain during severe NEC, ischemic brain damage due to conditions like shock, acidosis, and hypoxemia, and ongoing mucosal damage that hampers the absorption of essential nutrients.^118^ Future research should focus on understanding these mechanisms to develop targeted treatments that can reduce the neurological effects of NEC and improve the long-term outcomes for impacted infants.

## Microbial perspective

While the clinical perspective provides a clear depiction of the macro-level understanding of NEC, recent evidence suggests that a thorough comprehension of its pathogenesis necessitates a deeper understanding of the gut microbiota, a factor increasingly recognized for its significance in human health. Through a microbial perspective, we can delve deeper into the potential roles and characteristics of the microbial communities that underpin the development of NEC.

**Figure 2.**
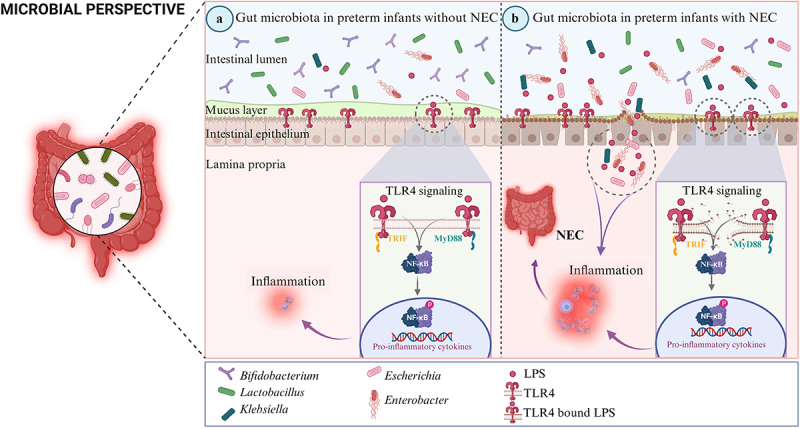
Microbial perspective of NEC. (a) Gut microbiota in preterm infants without NEC. In premature infants without NEC, beneficial bacteria such as the genera *bifidobacterium* and *lactobacillus* predominate, whereas potential pathogenic bacteria like *Klebsiella*, *Escherichia*, and *Enterobacter* are less prevalent. Under these conditions, LPS levels are lower, resulting in reduced TLR4 signaling and minimal inflammation. (b) Gut microbiota in preterm infants with NEC. In preterm infants with NEC, there is an increase in the abundance of potential pathogenic bacteria such as *Klebsiella*, *Escherichia*, and *Enterobacter*, together with a reduction in beneficial bacteria like *bifidobacterium* and *Lactobacillus*. This shift is associated with heightened production of LPS, which, upon interaction with TLR4, substantially activates the TLR4 signaling pathway. This in turn initiates a robust inflammatory cascade, predisposing to the development of NEC. In addition, a large number of potential pathogens can migrate to the intestinal lamina propria through a compromised intestinal barrier, which can also lead to gut inflammation, and, ultimately, the potential development of NEC.

In the human gut, a rich assortment of microorganisms cohabitate (termed as ‘microbiota’), forming a mutually advantageous alliance. It’s worth highlighting that the gut undergoes a notable ecological transformation when the infant transitions from the relatively sterile environment of the womb to the diverse microbiota present in their surroundings,^119^ and the neonatal period plays a vital role in the colonization of the gut microbiota, which is crucial for keeping the balance in the gut and ensuring overall well-being.^120,121^ The development of NEC is intricately linked to gut microbial colonization, given that NEC emerges post-colonization and can be mitigated in both human subjects and animal models through the administration of broad-spectrum antibiotics designed to target the enteric microbiota. Although the specific species or subspecies responsible for NEC are still not identified, a plethora of evidence points toward the crucial involvement of the gut microbiota in driving the onset of this condition (Figure 2).^122^

### Gut bacteriome

Early gut community perturbations pose the greatest risk for NEC.^123^ For healthy, full-term infants, the initial gut communities are dominated by *Bifidobacterium*, *Bacteroides*, *Escherichia*, and *Parabacteroides*.^124,125^ Subsequent colonization leads to a rapid increase in microbial diversity, with individual developmental trajectories influenced by various environmental factors such as diet, living conditions, and antibiotic exposure.^124,125^

In contrast, preterm infants have less diverse and distinct gut microbiota composition.^126,127^ The gut microbiota of these infants is mainly influenced by postmenstrual age and follows a characteristic progression with some variability.^128^ Initially dominated by bacilli after birth, transitioning to *Gammaproteobacteria*, notably *Klebsiella*, *Escherichia*, and other *Enterobacteriaceae*, then gradually shifting toward obligate anaerobic populations like *Clostridia* and *Negativicutes* in the absence of NEC.^122^ The rate of transition is accelerated in preterm infants with longer gestational periods, whereas anaerobic colonization is postponed to later stages of infancy in those with shorter gestational periods. This observation is significant in light of the timing of NEC development, as NEC tends to occur in the later stages of the neonatal period for preterm infants with shorter gestational periods.^21^

In preterm infants, the gut microbiota is predominantly composed of members from the Firmicutes and Proteobacteria phyla.^129^ Advances in metagenomic sequencing and 16S ribosomal RNA (rRNA) have revealed shifts within this microbial community in preterm infants with NEC, exhibiting diminished bacteria diversity. In addition, there is an increase in the abundance of Proteobacteria, particularly *Enterobacteriaceae* family, which is potentially pathogenic^122^; alongside a decrease in Firmicutes, specifically genera *Bifidobacterium* and *Lactobacillus*, which are known as beneficial bacteria.^129,130^ Numerous studies have corroborated similar findings, indicating that preterm individuals afflicted with NEC show marked dysbiosis in their intestinal microbiota.^131–133^ The main features are decreased diversity in the gut microbiota, a lack of beneficial commensal microbes, and the overgrowth of pathogenic bacteria.^134^ Notably, these disparities encompass an elevation in bacteria with the capacity to stimulate TLR4,^128,135^ which is recognized as a pivotal regulatory point in the inflammation process of NEC. In subsequent studies, a more important finding is that intestinal dysbiosis precedes the onset of NEC. The diversity of the microbiota before onset may serve as a potential risk factor for the condition. Consistently observed across preterm infant populations is the association between NEC and increased levels of Proteobacteria, specifically *Enterobacteriaceae*, alongside reduced levels of Firmicutes and Bacteroidetes before disease onset.^132,136^ While this pattern has repeatedly been documented, the specific genera within *Enterobacteriaceae* (*Klebsiella*, *Escherichia*, and *Enterobacter*) implicated in NEC may vary among different preterm cohorts. More intriguingly, Olm et al. observed a significant escalation in the replication rates of all bacteria, particularly the family *Enterobacteriaceae*, which are known to activate TLR4. This increase was notably pronounced two days preceding the diagnosis of NEC in preterm infants.^137^

Notably, multiple potential factors may be involved in the abnormal microbial colonization of the gut of preterm infants, leading to adverse outcomes including the development of NEC. For instance, prolonged use of antibiotics in early life, cesarean section (C-section) as a delivery method, and the absence of breast milk, etc. are all common factors that can significantly affect the gut microbiota of preterm infants.

#### Mode of delivery

Term infants born vaginally quickly acquire a beneficial gut microbiota from exposure to their mother’s gut and vaginal microbes at birth.^138,139^ In contrast, infants delivered via C-section – alters the initial vertical transmission – receive their initial gut microbiota from a variety of sources, such as the mother’s skin, the delivery room environment, and the microbiota of medical staff.^140^ C-section infants typically experience a persistent absence of *Bacteroides species* and delayed development of *Bifidobacterium*,^140,141^ while these are the main members of the early colonizers in the gut microbiota of infants born vaginally.^142–144^ Our meta-analysis of longitudinal metagenomic studies, using shotgun sequencing,^145^ indicated that infants born through vaginal delivery share a greater microbial species overlap with their mothers than those born via C-section. Although C-sections are typically performed on term babies, the rate of C-sections has increased worldwide in recent decades and remains important when it comes to preterm births, even reaching up to more than 50%.^146,147^ Notably, there is an ongoing debate in the scientific community and there is no consensus that the development of the gut microbiota in preterm infants is consistently related to the mode of delivery.^148–150^ However, the prevailing opinion is that this pattern in term infants does not apply to the development of the intestinal microbiota of preterm infants.^150–152^ The routine use of antibiotics in this group may mask the impact of birth mode on the gut microbiota. Once the effects of antibiotics have subsided, other factors – such as gestational age, physiological immaturity, and exposure to the neonatal intensive care unit (NICU) – become key drivers in the acquisition and assembly of the microbiota. In parallel, some studies have indicated that preterm infants delivered via C-section do not increase the risk of NEC.^153,154^

#### Feeding type

Feeding type is a key determinant that shapes the initial acquisition and subsequent colonization of the gut microbiota in neonates, affecting both preterm and term infants.^124,155^ In term infants, breastfeeding promotes a higher relative abundance of the *Bifidobacterium*—a beneficial gut commensal,^156^ while formula feeding is linked to increased levels of *Enterobacteriaceae*, *Bacteroidaceae*, and *Clostridiaceae*, potentially impacting gut health.^124,157^ In preterm infants, who have low bacterial diversity at baseline, non-breast milk feeds are associated with both a lower prevalence of *Bifidobacterium* and a reduced diversity of the gut microbiota,^158–160^ contrasting with the richer microbial community found in those fed with breast milk. Of note, the impact of breast milk on the microbiome of preterm infants is complex. For example, the gut microbiota of preterm infants could be influenced by host factors like gestational age, birth weight, postnatal time, and nutritional exposure. Interestingly, breast milk appears to mask the influence of birth weight, suggesting a dynamic interplay between the host and breast milk that facilitates the colonization and enrichment of specific microbes during the establishment of the gut microbiota in preterm infants.^161^ In preterm infants, feeding practice not only influences their gut microbiota but may also be associated with a range of health outcomes, including the risk of NEC, which is known to be reduced by breast milk.^162^ This beneficial effect of breast milk is attributed to bioactive components such as immunoglobulins, HMOs, lactoferrin, etc., all of which interact with commensal bacteria of the gut and play a crucial role in establishing the gut microbiota and ultimately influence intestinal inflammation in early life.^163^

Numerous studies have confirmed that receipt of maternal breast milk significantly reduces the risk of NEC among preterm infants, largely due to the high concentration of IgA antibodies – constituting over 90% of all antibodies – in breast milk.^164^ IgA, principally secreted by B lymphocytes, is abundantly present in the intestinal mucosa, where it defends against pathogen invasion and regulates the colonization of the gut microbiota.^81,164,165^ Since the development of intestinal B lymphocytes in newborns is not complete within the first 4 weeks of life, breast milk serves as the predominant source of their intestinal IgA.^81^ Breast milk-derived IgA binds to intestinal bacteria in offspring, forming IgA-bound bacteria. This process is one of the important mechanisms for shaping the colonization and composition of the gut microbiota.^81^ Normally, IgA-bound bacteria in the gut account for approximately 20–50% of the total microbial population.^81^ A deficiency in intestinal IgA or IgA-bound bacteria can lead to dysbiosis, lower microbial diversity, and increased prevalence of facultative anaerobic pathogens, such as *Enterobacteriaceae* and *Enterococcaceae*,^164,166^ The research by Gopalakrishna et al. indicated that breast-fed preterm infants harbor a greater abundance of IgA-bound bacteria than their formula-fed counterparts. Conversely, in NEC-afflicted preterm infants, the gut shows a higher proportion of IgA-unbound bacteria, especially *Enterobacteriaceae*, compared to preterm infants without NEC.^167^ However, in an animal model, mouse pups nursed by IgA-deficient mothers showed disease susceptibility akin to formula-fed peers, despite maternal milk exposure.^167^ This finding highlights the pivotal role of maternal IgA levels or the proportion of IgA-bound gut bacteria for the protection of NEC.

#### Antibiotics exposure

In preterm infants, antibiotic use is very common and sometimes prolonged, though not always indicated. Although antibiotics play a crucial role in preventing and treating life-threatening infections in this vulnerable population, they are also associated with severe morbidity, including NEC. Early use of antibiotics, particularly in the first two weeks of life, has been identified as a potentially important factor that may affect the likelihood of developing NEC.^168^ In a population-based retrospective study enrolling 4,932 VLBW infants, prolonged early antibiotic treatment (≥5 days) is associated with a more than twofold increased risk of developing NEC compared to short-term exposure (adjusted OR 2.27; 95% CI 1.02–5.06).^169^ Multiple retrospective studies have also confirmed similar results of higher NEC risk in the preterm population with longer early antibiotic exposure.^170^ Prolonged early antibiotics perturb the colonization of gut microbiota, leading to reduced microbial diversity, delayed commensal colonization, and overgrowth of pathogenic bacteria, a potential pathogenesis of NEC.^171^ Aberrant gut bacterial colonization, for example, beneficial bacteria like *Bifidobacterium* and *Bacteroides*, appear to be in lower abundance,^172^ while potentially pathogenic bacteria, such as *Enterobacteriaceae*, *Enterococcus*, and *Staphylococcus*, tend to be enriched.^173,174^ Furthermore, dysbiosis and fluctuations of the gut microbiome, resulting from this disruption, may persist for a long time even after discontinuing antibiotics.^175^ In the newborn mouse model, prolonged antibiotic exposure was found to impair intestinal development by reducing the proliferation of intestinal cells, the height of the villi, the depth of the crypt, and the number of Goblet and Paneth cells.^176^ This also explains why preterm infants who have been exposed to prolonged antibiotics after birth are more likely to develop NEC. Nevertheless, some researchers posit that early (within 2 to 3 days after birth) and short-term (3 to 5 days) antibiotic treatment, especially enteral antibiotics use, may reduce the incidence of NEC in preterm infants, also as supported by relevant animal models.^177–181^ However, this theory is not accepted in clinical practice and remains a contentious issue.

To reduce the risk of neonatal infections, especially *Group B Streptococcus*, prenatal antibiotics are commonly used for mothers. Maternal exposure to antibiotics may disturb the microbiota transmission from mothers to neonates.^182,183^ Specifically for preterm infants, antibiotic exposure (prenatal and postnatal), can influence the composition of the early gut microbiota. Compared to preterm infants with perinatal antibiotic exposure, those not exposed had a higher abundance of *Lactobacillus* in their meconium.^183^ In preterm infants with prenatal antibiotic exposure, the levels of *Bacteroidetes* and *Bifidobacterium* were substantially reduced at the 7th and 14th days postnatal, and the colonization of Bifidobacterium was delayed.^184^ Another finding from the study is that the initial gut microbiome of preterm infants exposed to antibiotics both in the prenatal and postnatal stages closely resembled the antibiotic-resistant bacterial profiles in the NICU during the corresponding period.

### Gut virome

Taken together, the integral role of gut bacteriome in the context of NEC has been in-depth researched, revealing consistent dynamic patterns. However, in addition to the gut bacteriome, the gut virome is gradually recognized as a critical component of the gut microbial ecosystem, significantly influencing the structure and function of gut bacteria.^185^ It is increasingly being viewed as a potential contributor to the etiology of NEC.^186^ The gut virome features a diverse array of bacteriophages (phages) that target gut bacteria, as well as eukaryotic DNA and RNA viruses infecting host cells throughout the gut.^187^ Notably, phages constitute the predominant component of this viral community. Similar to the bacteriome, the neonatal gut virome is virtually absent in the fetal gut but establishes itself rapidly within the first week after birth. The initial phage colonizers consist predominantly of induced prophages in the early gut bacterial colonizers,^187^ primarily derived from the maternal microbiota.^188^ Durante et al. identified vertical transmission of *Bifidobacterium spp*. containing prophages from breast milk to the gut of infants,^188^ suggesting a potential mechanism for the early establishment of the gut microbiota.

Olm et al. used next-generation sequencing (NGS) technology to analyze the gut microbiota of preterm infants, revealing an enrichment of certain phages, including *Staphylococcus* phage 363_30, before the onset of NEC.^137^ In a recent study of longitudinal fecal samples from 9 preterm infants with NEC and 14 gestational age-matched counterparts without NEC, a specific virome signature was identified, including reduced viral beta diversity and the presence of 137 viral contigs over the 10 days preceding NEC onset.^189^ Of the viral contigs that were linked to NEC and contained at least five open reading frames, nearly 70% were predicted to be lytic, while the rest were predicted to be temperate. In addition, in a study conducted by Cheng et al., involving 51 infants with NEC and 39 matched control infants without NEC, specific viruses such as enteroviruses and human bocaviruses were detected only in infants with NEC, and the presence of *adenoviruses* and *Epstein – Barr viruses* correlated with the severity of the disease.^190^ Furthermore, orogastric administration of fecal filtrate, containing pure virus-like particles, has been shown to completely prevent NEC in preterm piglets by increasing viral diversity and decreasing the relative abundance of Proteobacteria in the ileal mucosa.^98^ Such interventions restore the balance of the gut bacteriome and thus mitigate the severity of NEC. These results highlight the gut virome as an important mediator in the complex interplay between NEC and the gut bacteriome. This perspective is further substantiated by the latest empirical investigation demonstrating that dysbiosis of the gut bacteriome can result in the loss of intestinal epithelial cell resistance to autologous natural killer (NK) cell cytotoxicity, thereby inducing the onset of NEC during viral colonization or infection.^191^

## Molecular perspective

**Figure 3.**
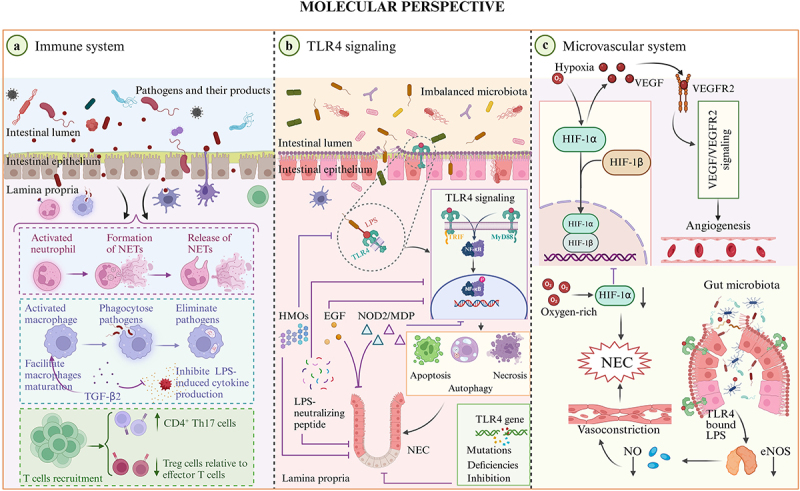
Molecular perspective of NEC. (a) Immune system. Neutrophils, rapidly migrate to sites of injury or infection upon the detection of tissue damage or the release of alarmins. They perform their function through the formation and release of NETs. In the context of NEC, the macrophage population increases in the intestinal tissue, where they play a crucial role in clearing bacteria that breach the intestinal epithelial barrier. TGF-β2 in the macrophage microenvironment enhances the inhibition of LPS-induced cytokine production and facilitates the maturation of immature macrophages. The immunomodulatory effect is correlated with a significant decrease in the incidence of NEC. In the intestine of NEC, T lymphocytes are extremely abundant, particularly CD4^+^ Th17 cells. These cells can induce a proinflammatory phenotype under appropriate conditions. In contrast, the proportion of anti-inflammatory regulatory T (Treg) cells relative to effector T cells is notably reduced, which is important in the pathogenesis of NEC. (b) TLR4 signaling. In the premature gut, TLR4 is upregulated, and an imbalanced microbiota activates TLR4 in response to dysbiosis (TLR4 recognizes its ligand, LPS) initiating a cascade of immunological and cellular events. Specifically, TLR4 signaling activation induces enterocyte demise through mechanisms of apoptosis, autophagy, and necroptosis. The cumulative impact of these pathological processes results in irreversible harm to the intestinal mucosa, a defining pathogenesis feature of NEC. Mutations, deficiencies, or inhibition of the TLR4 gene can confer protection against NEC. NOD2 and MDP (a NOD2 agonist) have a protective effect on NEC through the dampening of TLR4 signaling. HMOs can inhibit the LPS binding site on TLR4, which in turn reduces TLR4 signaling and consequently attenuates intestinal inflammation. EGF and LPS-neutralizing peptides, which inhibit TLR4 signaling, mitigate the pathological features of NEC. (c) Microvascular system. The relative hypoxia in utero serves as a potent stimulus for HIF-mediated signaling, which promotes VEGF synthesis, and activates VEGF/VEGFR2 signaling, culminating in the induction of fetal angiogenesis. Exposure to oxygen-rich conditions can result in supraphysiologic oxygen concentrations, leading to a reduction in HIF-mediated signaling, which may impair VEGF synthesis, and predispose premature infants to NEC. Intestinal mucosal damage enables bacterial translocation into the mesenteric microvasculature and triggers a cascade of interactions, notably with TLR4 on endothelial cells. This leads to a downregulation of eNOS and a subsequent reduction in nitric oxide production, which promotes vasoconstriction of the mesenteric vessels, and consequently intestinal ischemia, a hallmark of NEC.

Following the clinical and microbial perspectives, we further focus on changes at the molecular and cellular levels to uncover the deeper pathophysiological mechanisms of the disease, which may provide deeper insight into the rationale of clinical treatments and microbe-host interactions, facilitating the development of novel therapeutic strategies for infants with NEC in the future. NEC is a critical illness with complex molecular underpinnings, particularly in the realms of immune response, microvascular integrity, and cellular signaling. This section offers a detailed exploration of the molecular mechanisms driving the pathogenesis of NEC, focusing on as following: immune and inflammatory dynamics, VEGF-mediated microvascular regulation, and TLR4 signaling (Figure 3).

### Immune system and inflammatory mediators

The immune system is charged with the vital function of defending against invading pathogens. This process is contingent upon two pivotal components of defense strategy in the body: the innate immune system, which acts as a frontline defense against a broad spectrum of pathogens, including neutrophils, macrophages, dendritic cells, and others; and the adaptive immune system, which provides a targeted response to specific antigens, involving T lymphocytes, B lymphocytes, and their various specialized subtypes.^192,193^ In neonates, these defenses are immature, with a pronounced underdevelopment in preterm infants. This immaturity is presumably attributed to limited antigenic exposure, augmented autocrine and paracrine immune-suppressive pathways, weakened physical barriers, and diminished functionality among key cellular components.^194–197^ In addition, Toll-like receptors (TLRs) play an important role in the innate immune system by recognizing conserved molecular structures of pathogens, known as pathogen-associated molecular patterns (PAMPs).

#### Immune cells and inflammatory factors

Neutrophils, being the most abundant subset of the innate immune cell population, typically do not reside in healthy peripheral tissue. Instead, they rapidly migrate to sites of injury or infection upon the detection of tissue damage or the release of alarmins.^192^ These cells may perform their function through the formation and release of neutrophil extracellular traps (NETs), which are web-like structures containing enriched antimicrobial protein granules and neutrophil nuclear DNAs, aiding in the destruction of invading microorganisms.^198^ However, both neutrophils and NETs play a dual role in pathophysiology: they serve as beneficial agents in various processes when correctly localized and timed. However, excessive neutrophil activity or impaired clearance of NETs can potentially lead to tissue inflammation or damage.^198–201^

Empirical evidence from both human and murine ileal NEC tissue samples confirms the activation of neutrophils and the formation of NETs, illustrating the involvement of these processes in the pathogenesis of NEC.^202^ Collectively, these findings reveal the significance of neutrophil activation in the etiology of NEC. Nevertheless, conflicting data exist concerning the role of NET inhibition in NEC models. In a study by Chaaban et al.,^202^ the application of chloramidine, an inhibitor of NETs, to a murine model simulating NEC-like intestinal injury, resulted in exacerbated systemic inflammation, increased bacterial burden, heightened organ damage, and elevated mortality rates. Emami and colleagues demonstrated that the selective depletion of neutrophils and macrophages from the intestinal lamina propria was associated with the exacerbation of NEC, characterized by heightened inflammatory responses, villous atrophy, and enterocyte apoptosis.^203^ However, Klinke et al. reported that mice with a knockout of the *ELANE* gene, which encodes for neutrophil elastase (NE), resulting in a phenotype lacking in NE production – where markers of neutrophil activation and NET formation were undetected – presented protection against NEC, with an increased survival rate and minimal or no microscopic intestinal injury.^204^ Similarly, the daily subcutaneous injection of protein arginine deiminase 4 in NEC mice to inhibit NETs led to a significant reduction in NET production, tissue damage, inflammation, and mortality.^205^ The contradictions in these findings may be largely attributed to multiple factors, including variations in individual human samples with varying ages and NEC severities, the genetic lineage and age of the mouse strains at the time of NEC induction, the methods of NEC induction, and the timing of assessments.

Macrophages, integral to the innate immune system, are the predominant immune cells in the intestinal mucosa, where they protect the host from pathogens and contribute to intestinal homeostasis.^206^ Particularly in early life, they play a pivotal role in regulating intestinal immune defenses.^207^ In comparison to their wild-type peers infected with *Cronobacter sakazakii* (CS) – a pathogen implicated in NEC – macrophage-depleted mice presented aggravated inflammation, pronounced villous atrophy, and enhanced enterocyte apoptosis.^203^

In the neonatal intestine, macrophages are frequently replenished by blood monocytes that differentiate into intestinal lamina propria macrophages, which clear bacteria that breach the intestinal epithelial barrier.^208^ Notably, the ontogeny of neonatal intestinal macrophages is characterized by dynamically regulated immune defense functions. An *in vitro* study has shown that intestinal macrophages from fetal mice robustly express TNF-α upon LPS stimulation.^209^ In contrast, macrophages from neonatal mice display a heightened tolerance to LPS, suggesting a maturation-associated modulation in their inflammatory responsiveness. This phenomenon is linked to the rising levels of TGF-β in the macrophage microenvironment as pregnancy progresses, particularly TGF-β2, which enhances the inhibition of LPS-induced cytokine production in intestinal resident macrophages, typically maintaining a hypo-responsive state. Such a state is beneficial for fostering and sustaining a mutualistic relationship with the intestinal microbiota.^210^ In the context of NEC, there is an acute recruitment of blood monocytes to the damaged intestine, where they promptly differentiate into pro-inflammatory M1-type macrophages.^211^ Indeed, total and M1 macrophage populations were found to be increased in the intestinal tissue of NEC cases.^212^ M1 macrophages derived from both human NEC patients and animal models demonstrate elevated expression of *Smad7*.^213^ Subsequent study^209^ has proven that the enteral administration of TGF-β2 effectively facilitates the maturation of immature macrophages, which are initially dominated by pro-inflammatory phenotype, into a state with reduced inflammatory potential. The immunomodulatory effect of TGF-β2 is correlated with a significant decrease in the incidence of NEC, thereby highlighting its therapeutic potential in managing this severe neonatal condition. In addition, inhibiting the differentiation and effector functions of M1 macrophages is considered another approach to control the excessive inflammatory response in the NEC intestine.^214^

T lymphocytes, a central component of the adaptive immune system, are implicated in NEC through their diverse roles. Previous authors have shown that in the small intestine of both human and mouse NEC, T lymphocytes are extremely abundant, particularly CD4^+^ Th17 cells.^92^ These cells can induce a proinflammatory phenotype under appropriate conditions, such as the presence of specific cytokines, tissue damage, or other immunological signals. In contrast, the proportion of anti-inflammatory regulatory T (Treg) cells relative to effector T cells is notably reduced, which is important in the pathogenesis of NEC.^92,215^ Flow cytometry analysis of T cells within the lamina propria, derived from ileal samples of 18 preterm infants with NEC and 30 without NEC, revealed a higher prevalence of Treg cells in the NEC cohort. Nonetheless, the ratio of Treg cells to both CD4 and CD8 T cells was considerably reduced in the NEC group. Concurrently, increased gene expression levels of IL-1β, IL-6, IL-8, IL-10, and TNF-α were observed, suggesting an inhibitory effect of inflammatory cytokines on Treg development, a potential factor in NEC pathogenesis.^215^

Importantly, infiltrating pro-inflammatory T cells are primary instigators in NEC development.^216^ Evidence suggests that the adoptive transfer of mucosal CD4^+^ T cells, activated by NEC, into naive mice spontaneously induces intestinal inflammation.^92^ However, lymphocyte-deficient *Rag1*^–/–^ mice, lacking both T and B cells, are protected from NEC development. Strategies that block the receptor for IL-17, which is a pro-inflammatory cytokine produced by CD4^+^ Th17 cells, attenuate NEC severity. Interestingly, differences in immune profiles between preterm and term infants have been reported to become more pronounced as they mature, leading to a net effect of a skew toward a pro-inflammatory state in the preterm infant population.^217^ This tendency is also reflected in the premature intestinal epithelium, which is marked by a proinflammatory microenvironment that has been noted previously.^218,219^

#### TLR4 signaling

TLRs are a family of pattern recognition receptors (PRRs). TLR4, in particular, is instrumental in the recognition of lipopolysaccharide (LPS), a component of the outer membrane of Gram-negative bacteria, which can trigger a robust inflammatory response.

Emerging research suggests that TLR4 is upregulated in the intestinal mucosa of preterm infants compared to full-term infants across multiple species.^62,92,220^ This finding is significant as TLR4 signaling plays a crucial role in regulating gut development by interacting with the Wnt and Notch signaling pathways, which are essential for maintaining stem cell niches in the small intestine.^220–222^ The interplay between these pathways is essential for the proper differentiation and proliferation of intestinal stem cells, highlighting the critical role of TLR4 in the intricate coordination of intestinal morphogenesis and immune homeostasis. However, the role of TLR4 during in-utero development transforms the context of preterm birth, potentially transitioning from a facilitative to a detrimental function. In particular, prolonged upregulation of TLR4 can lead to pathological consequences through activation by luminal bacteria containing TLR4 ligands such as LPS.^130^ This interaction has the potential to induce mucosal damage and contribute to the progression toward intestinal ischemia and the subsequent onset of NEC. These findings underscore the necessity for a nuanced understanding of TLR4’s dualistic role and its complex interplay with the premature intestinal ecosystem. Meanwhile, the ‘TLR4 switching’ paradigm offers crucial mechanistic perspectives on the pathogenesis of NEC, partially elucidating the increased susceptibility of preterm infants to NEC as compared to those born at term.^223^

A substantial body of evidence supports a pathogenic mechanism model for NEC that is predicated on the pivotal role of the LPS receptor, TLR4. Following a meticulous analysis of the transcriptional profiles of both human and murine intestines afflicted with NEC, a marked upregulation of TLR4 was identified.^224^ Concomitantly, an analogous increase in TLR4 expression was observed in rats exhibiting NEC,^225^ underscoring the conserved role of TLR4 in the pathophysiological response across species.

In the premature gut, characterized by an imbalanced microbiota, the activation of TLR4 in response to dysbiosis initiates a cascade of immunological and cellular events. Specifically, TLR4 activation induces enterocyte demise through mechanisms of apoptosis,^226–228^ autophagy,^229–231^ and necroptosis,^232^ concurrently hindering mucosal repair^233,234^ and cellular proliferation.^221,222^ The cumulative impact of these pathological processes results in irreversible harm to the intestinal mucosa, signifying a defining feature of NEC’s pathogenesis. Seminal research has shown that mutations, deficiencies, or inhibition of the TLR4 gene can confer protection against NEC.^62,220,235,236^ In parallel, the TLR4 signaling pathway has been established as a significant contributor to the pathogenesis of NEC. In an important human study conducted by Sampath et al., it was revealed that mutations in the single immunoglobulin IL-1-related receptor (SIGIRR) gene, an important negative regulator in the TLR4 signaling pathway,^237^ are prevalent in a substantial number (10/17) of individuals with severe NEC.^238^ Richardson et al. demonstrated that nucleotide-binding oligomerization domain-containing 2 (NOD2),^70^ an intracellular pattern recognition receptor, can inhibit TLR4 activation in the gut, thereby preventing NEC. This protective effect is mediated through the recognition of muramyl dipeptide (MDP) by NOD2. Accordingly, administering MDP, acting as a NOD2 agonist, to mice has been shown to reduce the severity of NEC through the dampening of TLR4 signaling.^70^ Moreover, Chan et al. found that an LPS-neutralizing peptide, which inhibits TLR4 signaling, mitigates the pathological features of NEC in the rat jejunum.^225^ Intriguingly, a study by Klerk et al. revealed that human DNA extracted from the fecal samples of preterm infants exhibited increased TLR4 methylation in those diagnosed with NEC,^239^ thereby providing further evidence for the involvement of TLR4 in the pathogenesis of the disease. In addition, oligosaccharides in breast milk can inhibit the LPS binding site on TLR4, which in turn reduces TLR4 signaling and consequently attenuates intestinal inflammation.^240^ Furthermore, epidermal growth factor (EGF), another important component of breast milk, suppresses TLR4 signaling in the premature gut of mice and cultured enterocytes, which is achieved by reducing TLR4-induced NF-κB through EGF-mediated activation of the PI3K-AKT pathway.^84^ In a striking parallel to breast milk, amniotic fluid, known for its high concentration of EGF,^241^ inhibits TLR4 signaling by binding to EGF receptors on the intestinal epithelial cells.^242^ These provide additional support for the role of TLR4 in the development of NEC, and in parallel, reveal the preventative and therapeutic potential of breast milk and amniotic fluid in the context of NEC.

Although most studies affirm the link between TLR4 activation and NEC progression in preterm infants, some authors provide an alternative perspective, proposing that TLR4 expression in the NEC model does not consistently increase. An earlier study using the piglet NEC model showed no increase in TLR4 expression in the intestine.^243^ Subsequently, White et al. developed an NEC model for mice in which Paneth cells are disrupted, which occurs independently of TLR4 signaling, as TLR4-deficient (*TLR4*^*−/−*^) mice presented a similar severity of NEC as wild-type mice.^244^ However, in the later study, the NEC model was induced at an older age (P14-P16), a stage at which TLR4 levels in the gut were quite low.^62^ Nevertheless, these findings suggest that the role of TLR4 in NEC may be more complex than initially thought. Therefore, further clinical and laboratory studies are warranted to better understand this relationship.

In addition, TLR4 is intimately involved in the complex interplay with immune cells and inflammatory factors. In preterm infants, heightened expression of TLR4 in the intestinal epithelium triggers upregulation of the lymphocyte chemoattractant CCL25, leading to the recruitment of naive T cells to the intestinal lamina propria.^92^ This environment, characterized by elevated levels of proinflammatory cytokines such as IL-6 and IL-22, facilitates the differentiation of T cells into Th17 cells, which is crucial for NEC development. In this process, the upregulated transcription factor RORγt plays a pivotal role in promoting Th17 cell development. Additionally, TLR4 mediates STAT3-dependent polarization, where an increase in pSTAT3 levels elevates the expression of RORγt and reduces the induction of Foxp3—a transcription factor essential for Treg cell differentiation – ultimately driving T cells toward a Th17 phenotype.^92^

### Microvascular system and VEGF signaling

The growing evidence shows the essential role of the intestinal microvasculature in both the initiation and progression of NEC, highlighting its status as a central mechanism in the pathogenesis of this disease.

VEGF-A, an integral component of the VEGF family, plays an indispensable role in angiogenesis and the development of embryonic vasculature, with its signaling primarily transduced via the receptor, VEGFR2.^245^ In murine models, VEGF and its receptor VEGFR2 exhibit heightened expression in the intestinal tissue during the final three days of gestation, specifically around the 20-day mark, which is critical for the proper maturation of the intestinal microvasculature immediately before parturition.^246^ This process is meticulously regulated by hypoxia-inducible factor-1 (HIF-1), which serves as a master regulator in responding to hypoxic conditions and coordinating the transcription of genes essential for fetal growth and vascular development.^247,248^ In neonatal mice, treatment with dimethyloxalylglycine (DMOG) – a stabilizer of HIF-1α—reduced the severity of NEC, enhanced intestinal VEGF expression, and promoted the proliferation of villus endothelial and epithelial cells in a VEGFR2-dependent manner.^249^

The relative hypoxia in utero serves as a potent stimulus for fetal angiogenesis. However, the oxygen-rich extrauterine environment, even with just room air at 21% oxygen, surpasses the oxygen levels in the womb. Exposure to such conditions, whether through therapeutic oxygen supplementation or simply due to the ambient air, can result in supraphysiologic oxygen concentrations, leading to a downregulation of HIF and its downstream targets.^248^ The consequent reduction in HIF-mediated signaling, particularly during the critical period of intestinal vascularization, may impair VEGF synthesis. This disruption in VEGF availability can predispose extremely preterm infants to NEC. A finding revealed that HIF-1α expression peaks in the most damaged NEC tissues, which lack microvasculature, in stark contrast to the less damaged or unaffected ileum.^250^ This correlation suggests a link between NEC, hypoxia, diminished endothelial cell count, and reduced microvessel density.

Intestinal VEGF/VEGFR2 signaling defects can reduce vascular density and endothelial proliferation, increasing the susceptibility of infants to NEC. Pre-treatment of newborn mouse pups with VEGFR2-specific kinase inhibitors before NEC induction resulted in elevated mortality, increased severity of intestinal necrosis, and reduced intestinal microvascular network density in comparison to untreated controls.^246^ Additionally, the literature revealed that pups exposed to NEC stressors showed a decreased intestinal microvascular network density compared to dam-fed controls, with the phenotype being exacerbated by the concurrent blockade of VEGFR2 signaling. In human studies, similar patterns were noted. A clinical trial involving 128 VLBW infants and 200 healthy term neonates showed that VLBW infants had a higher occurrence of the VEGF + 405C allele compared to the healthy group.^251^ Meanwhile, the VEGF-2578A allele, which is linked to reduced VEGF gene expression, was identified as an independent risk factor for NEC. Examination of intestinal villi in NEC patients undergoing surgery revealed a significantly lower count of VEGF-A+ cells compared to infants with other intestinal conditions.^252^ Moreover, it is widely recognized that intrauterine inflammation, exemplified by chorioamnionitis, is associated with an elevated risk of NEC. Such prenatal inflammation is implicated in the disruption of intestinal microvasculature development through the downregulation of VEGF and VEGFR2 signaling.^253^ In contrast, the experimental overexpression of VEGF in rat models resulted in notable mitigation of villous atrophy and tissue edema,^254^ indicating a potential therapeutic role for VEGF in modulating intestinal mucosal integrity.

Interestingly, researchers have discovered that the gut microbiota is essential for the development of postnatal intestinal angiogenesis. Germ-free mice have been observed to possess an immature intestinal capillary network when compared to animals with functional microbiota.^255^ However, intestinal mucosal damage, a critical event in the pathogenesis of NEC, enables bacterial translocation into the mesenteric microvasculature.^256^ This process triggers a cascade of interactions, notably with TLR4 on endothelial cells. The engagement of TLR4 leads to a downregulation of eNOS, an enzyme integral to nitric oxide synthesis.^77^ Consequently, the reduced production of nitric oxide – a key vasodilator – facilitates the mesenteric vessels to vasoconstriction, thereby promoting the intestine to ischemia, a hallmark of NEC.^77^ These findings illustrate the critical interplay between the intestinal microbiota and the immature vascular endothelium in impairing intestinal perfusion, a significant factor in the progression of NEC.

## Conclusions and future perspectives

NEC is a complex disease that remains a leading cause of death and disability among the vulnerable population of preterm infants. Although there is still no specific treatment for NEC, significant progress has been made in the last decade. Findings from clinical observations, microbiological analysis, and molecular research have collectively improved our understanding of the mechanisms underlying the pathogenesis of NEC. Future research in the field of NEC in preterm infants should focus on elucidating the specific role of pathogenic metabolic pathways in the pathogenesis of NEC, applying advanced artificial intelligence techniques such as machine learning to improve the accuracy of early diagnosis, further explore the TLR4-dependent signaling pathways that drive NEC progression, and develop targeted strategies to mitigate the range of long-term morbidities associated with NEC, thereby improving both acute clinical management and long-term health outcomes for these vulnerable infants. With these targeted research initiatives, we aim to refine diagnostic precision and broaden therapeutic options for NEC.
